# Scaling behavior and text cohesion in Korean texts

**DOI:** 10.1371/journal.pone.0290168

**Published:** 2023-08-31

**Authors:** Hokyun Kim, Sanghu Park, Minhyuk Jeong, Hyungi Byun, Juyub Kim, Doo Yong Lee, Jooyoung Jeon, Eojin Yi, Kwangwon Ahn

**Affiliations:** 1 Korea Advanced Institute of Science and Technology, Moon Soul Graduate School of Future Strategy, Daejeon, South Korea; 2 Department of Industrial Engineering and Center for Finance and Technology, Yonsei University, Seoul, South Korea; 3 FNC Technology Co., Ltd., Gyeonggi-do, South, Korea; 4 Seoul Business School, aSSIST University, Seoul, South Korea; University of Sao Paulo, BRAZIL

## Abstract

This study examines whether different types of texts, particularly in Korean, can be distinguished by the scaling exponent and degree of text cohesion. We use the controlled growth process model to incorporate the interaction effect into a power-law distribution and estimate the implied parameter explaining the degree of text cohesiveness in a word distribution. We find that the word distributions of Korean languages differ from English regarding the range of scaling exponents. Additionally, different types of Korean texts display similar scaling exponents regardless of their genre. However, the interaction effect is higher for expert reports than for the benchmark novels. The findings suggest a valid framework for explaining the scaling phenomena of word distribution based on microscale interactions. It also suggests that a viable method exists for inferring text genres based on text cohesion.

## 1. Introduction

The English word “text” originates from the Latin word “*texere*,*”* which means “to weave.” The written text was a metaphor for a complex structure of ideas woven together to form a singular, complete inscription [1]. Text is conceptually complex, corresponding to its etymological origin. The exact dynamics of the factors within the text production and the influence of individual factors on the entire structure remain unknown. As each piece of text is unique and complex, it is intractable to classify text according to every attainable feature. However, this task is feasible by aggregating and organizing multiple groups of text with similar text attributes [2], allowing huge amounts of text to be handled and understood.

It is well documented that word frequencies in natural language follow a power-law distribution with an exponent approximately to unity, i.e., Zipf’s law. However, the underlying dynamics and origin of Zipf’s law in word frequencies are still unclear. This is not because no valid theoretical explanation induces Zipf’s law in the creation of natural language. Rather, it is because there are too many valid explanations [3–6] as the power-law can be derived in many different ways [7]. Particularly, Piantadosi [8] argued that a model’s ability to induce Zipf’s law is insufficient. For a certain model to be credible as a viable explanation to connect natural language and Zipf’s law, it must be able to properly refer to the cognitive and psychological process from which text is produced.

One factor affecting word distributions of different types of texts is the choice of language. Like any other linguistic unit, language can be classified according to its characteristics and structural features. The classification of different languages is called language typology. Language can be classified according to different standards from different fields of linguistics, such as phonetic, morphologic, and syntactic. Our study is directly related to the formation of each word; therefore, we approach language morphology from a morphological perspective. Specifically, we compare Korean text with text from other languages and employ the scaling exponent in word frequencies as lexical cues, indicating which morphological language typology each text belongs to.

Other factors influencing word distribution include the context in which the text was written, the genre which the text belongs to, or the author’s writing style. Although these aspects affecting each piece of text are often overlooked, Baayen [9] demonstrated how numerous text-related factors, including genre, writing style, or context, can affect word distributions. In this study, we use the scaling exponent to compare how word distributions are expected to behave according to a particular factor, “genre.”

Specifically, we investigate word distributions of two types of Korean natural languages: event reports in nuclear power plants and a novel as a benchmark. Then, we test the hypothesis that the event report on the nuclear power plant, one of the formal and official documents, will show stronger text cohesion than our benchmark, which is a piece of literature that matches the size of the document. We measure the rate of text cohesion according to the interaction effect, which is deduced by introducing the controlled growth process model [10] with empirical word distribution. Finally, we compare the interaction effects in two documents to analyze the impact of text cohesion in different genres.

Section 2 describes sample texts and explains their preprocessing. Section 3 introduces the theoretical framework nesting the scaling exponent of a word frequency distribution in a closed-form solution. Section 4 discusses the scaling behavior in different languages and its implied interaction effect in different text genres. Finally, Section 5 presents the conclusion and future studies.

## 2. Materials

### 2.1. Data description

A nuclear power plant incorporates the most complex and delicate technologies known to humankind. Because safety is important in a nuclear power plant operation, experts meticulously draft event reports. We collected 1,097 documents of structured event reports related to nuclear power plants provided by the Korea Institute of Nuclear Safety. The styles of the documents range from handwritten to word processor-printed. Among these reports, we selected four reports (Report no. 1–4) with comparable page lengths and vocabulary sizes for our analysis. We have examined other technical reports on nuclear power plants but most of them are either too short or not publicly available. This is because most information on nuclear power plants is strictly secured by government agencies, such as the Nuclear Safety and Security Commission and Korea Institute of Nuclear Safety. Three Korean novels titled “*Mu-Myeong*,” “*Choe-Hu-Ui-Ak-Su*,” and “*Dong-Eop-Ja*” were chosen as benchmarks. Specifically, we choose copyright-free novels according to Berne Convention for the Protection of Literary and Artistic Works [11]: all works must be protected for 50 years after the author’s death. The sources of all the documents we have used are given in S1 Appendix. We assumed our selected novels to represent Korean documents that adhere to what could be regarded as general discourse.

Table 1 summarizes the statistics of our sample texts. Here, *V* represents the vocabulary size, which is set within a certain threshold to match the relative size of each document (*V* ≥ 1,000); *n*_*max*_ represents the maximum word frequency occurring in each document; *a* represents the minimum word frequency ensuring that the power-law assumption holds in the word distribution; *N*_*a*_ represents the number of different words considered in a power-law fitting (*n* ≥ *a*), which refers to all words accounted for estimating the scaling exponent. Clauset et al. [12] proposed a method for determining the cutoff point *a*: Cutoff point *a* is estimated by minimizing the distance between the empirical cumulative density function and the theoretical value using Kolmogorov–Smirnov (KS) statistic. Specifically, for each possible cutoff point, scaling exponent is estimated via maximum likelihood estimator with observations whose frequencies are larger than the cutoff point, then the most plausible cutoff point *a* is determined according to the KS goodness-of-fit statistics. As the *a* values of the three Korean novels are equal to 2, words occurring only once are excluded from their power-law fitting. Likewise, words whose frequencies were below their cutoff points are excluded in the calibration process for estimating the parameters for the controlled growth process model.

**Table 1:** Summary of the sample texts.

Title	# of words	*V*	*n*_*max*_	*N_a_*	*a*
Report no. 1	4,085	1,365	133	1,365	1
Report no. 2	3,271	1,264	178	1,264	1
Report no. 3	2,821	1,026	123	1,026	1
Report no. 4	2,787	1,053	77	1,053	1
*Mu-Myeong*	15,405	4,685	674	1,399	2
*Choe-Hu-Ui-Ak-Su*	17,454	4,881	644	1,562	2
*Dong-Eop-Ja*	5,731	2,201	200	576	2

Note: Compared to other documents, novels exhibit relatively large sizes of vocabulary size. This can be interpreted as a lexical cue, as such word distribution concurs with the conventional perception that words occurring sporadically are more likely to instantiate in novels as opposed to nuclear event reports, increasing the relative volume of *hapax legomena* (words occurring only once).

### 2.2. Data preprocessing

Recently, the amount of unstructured data has increased exponentially. Extracting meaningful information from such a vast amount of unstructured information requires analyzing large pieces of data that have been drastically accelerated in tandem with technological advances. Artificial intelligence and neural networks [13] combine to make this approach implementable. This operation can be simplified into two parts: coding and parsing the data according to linguistic units. The classification process becomes more concise by parsing the data and setting the criterion to concrete values, such as word frequency, allowing large datasets to be evaluated [2].

**Fig 1:**
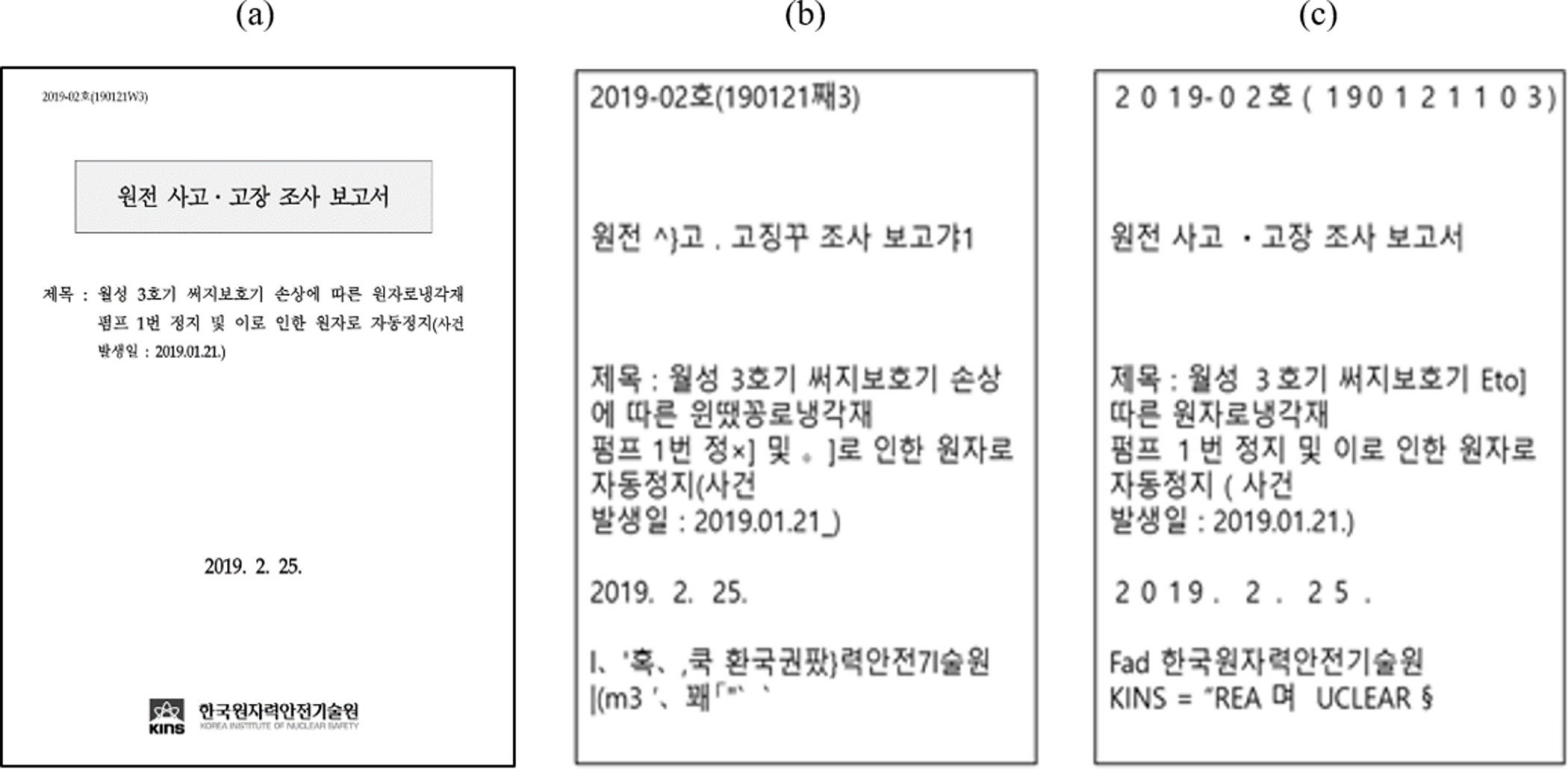
Performance of OCR engines on the cover page of an event report. The images provided are (a) the original cover page, (b) OCR results by the legacy engine, and (c) OCR results by the LSTM engine. The LSTM engine shows 18%p higher accuracy than the legacy engine. The extraction accuracy is measured according to each Korean character, defined as a morpho-syllabic block. Each block is constructed as a combination of i) a single vowel with the Korean null initial consonant “ieung” used as a placeholder, ii) an initial consonant and vowel, or iii) an initial consonant, a vowel, and a final consonant.

Specifically, we extract text from the image file of each document using Tesseract 5.0, an open-source optical character recognition (OCR) engine. Tesseract 5.0 supports a legacy model that proceeds with a traditional step-by-step approach based on feature extraction and hierarchical shape classification [14]. It also supports a long short-term memory (LSTM) neural network model. The performance of both OCR engines was compared using the accuracy scores measured by the character error rate and Levenshtein distance [15]. Fig 1 shows an example. As LSTM engines perform better on our data than the legacy engines, we used the Tesseract 5.0 OCR engine based on LSTM models to convert the documents to analytical forms.

Text extracted with the OCR engine must be preprocessed into word form. Unlike most languages, where words can be parsed according to blank spaces and punctuation marks, certain function words in Korean immediately follow and fuse to nouns and pronouns. Each text component was grammatically annotated by morpheme analysis to detect and distinguish such function words. The analyzed text was parsed into words and placed in a data table according to frequency.

## 3. Theoretical framework

### 3.1. Controlled growth process model

Many previous studies explained how exogenous factors could affect word distribution [9] and investigated the word distribution on syntactic and semantic dimensions [16]. However, most studies associating natural language with Zipf’s law by modeling the word distribution fail to explain the origin of their emergence in the form of a theoretical perspective. Instead, they focus on reporting that certain text or corpus follows Zipf’s law from an empirical perspective. Therefore, we propose a theoretical framework that incorporates uniform-size production into the process of emerging each piece of text. Consequently, we obtain a closed-form solution that nests the interaction effect in the scaling exponent.

We employ the approach introduced by Kim et al. [10] to model the evolution of the word distribution. We consider the word frequencies to be evolving elements forming a probability distribution. The frequency *x* of a word grows to *x’* when it appears on the following unit progression; we refer to this as an event. Accordingly, the amount of growth, Δ*x* ≡ *x’ ‒ x*, depends on the current value *x*. Based on the Yule process, we define that Δ*x* is proportional to *x*, i.e., Δ*x* = *bx*, and describe the transition rate associated with an event in the following form

$$\omega \left(x\to x^{\prime }\right)=\lambda \delta \left[x^{\prime }-(1+b)x\right],$$ (1)

where *λ* and *b* are the occurrence rate of events and growth factor, respectively; and *δ* is the Dirac delta function.

Suppose that there are *N* different words up to a given progression *t* and the frequencies of the *N* different words, *x*_*i*_ for *i* ∈ {1,2,…,*N*}, have the probability *P* (*x*_1_,…,*x*_*N*_;*t*). The probability *P* (*x*_1_,…,*x*_*N*_;*t*) is related to the distribution function *f* (*x*, *t*), which can be expressed as

$$f(x,t)=\frac{1}{N}\int dx_{1}\cdots dx_{N}\sum_{i}\delta \left(x_{i}-x\right)P\left(x_{1},\ldots ,x_{N};t\right).$$ (2)

Note that each frequency value can change according to the transition rate in Eq (1). Then, the time evolution of the probability *P* (*x*_1_,…,*x*_*N*_;*t*) can be conveniently described by a master equation [17]. Specifically, the master equation reduces to the evolution equation given as

$$\frac{\partial }{\partial t}f(x,t)=-(r+\lambda )f(x,t)+\frac{\lambda }{1+b}f\left(\frac{x}{1+b},t\right)+rg(x,t),$$ (3)

where *g*(*x*,*t*) is the distribution function of newly produced word frequencies with the production rate *r*. Production of a new word is referred to as the emergence of the first word on a given progression *t*.

Each progression of a certain document is expected to have a new word whose frequency may vary within a limited range; thus, it is more or less uniform. In such a case of uniform-size production, i.e., *g* (*x*, *t*) = δ(*x* ‒ *x*_0_), the power-law type distribution provides the stationary-state solution of Eq (3) [10,18]. Then, the scaling exponent *α* of this model depends on the parameters as follows:

$$\alpha =\frac{\text{ln}(1+r/\lambda )}{\text{ln}(1+b)}.$$ (4)

The model is further extended to incorporate the interaction effect *c*_*ij*_ within the words constituting the text. We assume that there is a tendency to reduce the frequency difference between words, leading to the contribution *c*_*ij*_(*x*_*j*_ ‒ *x*_*i*_) of the *j*th frequency to the amount of growth of the *i*th frequency. If detailed information about the interaction effect is not available, it is rational to reduce bias as much as possible. Thus, we use the uniform interaction effect *c*_*ij*_ = *c*/*N*, which makes the least biased presumptions and corresponds to the mean-field theory in physics [19]. Then, we sum *c*_*ij*_ over *j* to obtain Δ*x*_*i*_ = *bx*_*i*_+*c$\bar{x}$*, where *c*/*N* has been absorbed into *b*; thus, $\bar{x}\equiv N^{-1}\sum_{j(\neq i)}x_{j}$ essentially becomes the average word frequency. Consequently, Eq (1) changes to

$$\omega \left(x_{i}\to x_{i}^{\prime }\right)=\lambda \delta \left[x_{i}^{\prime }-(1+b)x_{i}-c\bar{x}\right].$$ (5)

Finally, the scaling exponent in Eq (4) is revised as follows:

$$\alpha_{int}=\frac{\text{ln}\left(1-c^{2}/2\right)+\text{ln}(1+r/\lambda )}{\text{ln}(1+b)}.$$ (6)

### 3.2. Power-law in a word frequency distribution

The scaling exponent *α* of a word frequency distribution can be directly obtained from the number of different words with a given frequency in a text. Specifically, the distribution function *f* (*x*_*i*_) that a word has frequency *x*_*i*_ is given by

$$f\left(x_{i}\right)\propto \frac{1}{x_{i}^{\alpha +1}},$$ (7)

where *x*_*i*_ is the frequency of a word *i*.

The other way of expressing the scaling exponent *ζ* is from the frequency *x*_*i*_ of the *R*-th most frequent word of a text

$$x_{i}(r)\propto \frac{1}{R\zeta },$$ (8)

where *R* is the rank of each word.

The real values of *f* (*x*_*i*_) and *x*_*i*_ (*R*) contain the information about the word’s frequencies in a text; however, *f* (*x*_*i*_) does it in a compressed fashion [20]. The two scaling exponents *α* and *ζ* are related as follows:

$$\alpha =\frac{1}{\zeta }.$$ (9)

Therefore, if a word frequency distribution follows Zipf’s law, *α* and *ζ* should be close to unity.

### 3.3. Estimation strategy

It is well-known that a word frequency distribution of natural language follows Zipf’s law [8,21]. However, only a few empirical phenomena follow a power-law type distribution for the entire range of observations. Moreover, it is common to consider only values that are greater than a certain minimum value when fitting the power-law distribution [12]. Accordingly, we estimate *α* for the selected texts with observations whose frequencies are larger than the cutoff point *α* using a maximum likelihood estimator [12]. Meanwhile, when comparing scaling behavior in different languages or genres, the exponent *α* is converted into *ζ* from Eq (9) because most previous studies examined scaling behaviors of word frequencies in the form of *x*_*i*_(*R*).

We calibrate the parameters of the controlled growth process model directly from the data to compare the text cohesion in each document. We employ the idea introduced by Choi et al. [17] to calculate the growth factor as *b* = (*x*′ ‒ *x*)/*x* and the occurrence rate as *λ* = *l*/*N*, where *l* is the number of updated words occurred in each unit of progression. Moreover, the production rate is calibrated from *dN*/*dt* = *rN*. The median of each calculated value, e.g., *b* and *λ*, is taken for each document. The slope of the fitted line for log *N* to *t* is taken for *r*. Details about the calibration processes of *b*,*λ*, and *r* are presented in S2 Appendix.

Considering the interaction effect in the controlled growth process model, we calculate the interaction effect among words using the calibrated parameters (*b*, *λ*, and *r*) and scaling exponent, which is directly estimated from the word frequency distribution, namely *α*_*emp*_. Specifically, *α*_*emp*_ is assumed to be the same as the scaling exponent derived from the controlled growth process model with (uniform) interaction effects; that is, we set *α*_*emp*_ = *α*_*int*_. Then, the interaction effect *c*, used as a proxy for text cohesion, can be calculated using Eq (6). In this context, we henceforth notate our estimate of the interaction effect as the *implied* interaction effect.

## 4. Results and discussion

### 4.1. Scaling behavior in different languages

Table 2 presents the scaling exponents for different languages reported by previous studies. Choi [22] investigated whether the scaling exponents are consistent across languages; particularly, for Korean, English, and French, each value was estimated as *ζ* = 0.9, 1, and greater than 1, respectively. Choi [22] also evaluated scaling exponents for Korean corpora with varying corpus sizes. The findings showed the scaling exponent values were consistent within the range of 0.89 and 0.94, regardless of the corpus size. Corral et al. [20] reported that word distributions in Finnish novels, which morphologically belong to the same linguistic typology as Korean, display scaling exponents near the range of scaling exponents of Korean novels. Thus, the word distributions of Korean and Finnish novels do not follow Zipf’s law. This can be distinguished from scaling exponents of English and French texts whose scaling exponents are near or greater than 1 [20,23,24].

**Table 2:** Scaling exponents for different languages.

Language	*ζ*	Text	Reference
English	1.20	Clarissa	Corral et al. [20]
	1.11	Moby-Dick	Corral et al. [20]
	1.03	Ulysses	Corral et al. [20]
French	1.02	*Google* 1-gram dataset (1800–2008)	Petersen et al. [23]
	1.16	Holy Bible	Mehri et al. [24]
Finnish	0.88	Seitsemän	Corral et al. [20]
	0.95	Kevät ja	Corral et al. [20]
	0.92	Vanhempieni	Corral et al. [20]
Korean	[0.89,0.94]	KAIST corpus	Choi [22]

As presented in Table 3, Korean texts do not follow Zipf’s law as their scaling exponents, *ζ*, are less than 1. Therefore, the scaling behavior of Korean and English word frequencies differs, which could be because of the morphological difference between languages. Unlike English, Korean is agglutinative, meaning it has many combinations of postpositions that can be appended to the root of a predicate [22]. Low scaling exponents can also be found in documents in Finnish, which is a typical agglutinative language. In other words, language typology could be directly associated with Zipf’s law, as languages morphologically categorized as agglutinative are structurally more apt for word-formation. Thus, they are expected to display a richer vocabulary, resulting in a smaller scaling exponent than languages from other morphological categories.

**Table 3:** Scaling exponent of each text belonging to the different genres in Korean texts.

Title	$\hat{\zeta }$ ±*SE*_ζ_
Report no. 1	0.91 ± 0.03
Report no. 2	0.80 ± 0.03
Report no. 3	0.85 ± 0.04
Report no. 4	0.87 ± 0.04
*Mu-Myeong*	0.92 ± 0.03
*Choe-Hu-Ui-Ak-Su*	0.93 ± 0.03
*Dong-Eop-Ja*	0.83 ± 0.05

Note: The standard error (SE) of each document is determined as $\hat{\zeta }{\left(N_{a}/2\right)}^{-\frac{1}{2}}$ [25].

### 4.2. Scaling behavior in different text types

Many previous studies that classified text types based on word frequency could be divided into two categories. One category consists of using lexical cues, such as the frequency of punctuation marks as signatures or the frequencies of a selective set of words to distinguish different text types [26,27]. The other category consists of methods assigning word frequency of each term occurring in all documents on a document-term matrix and then conducting principal component analysis on the matrix, expecting to capture latent features through dimensional reduction [28,29]. This method was developed for topic models, which assign each document to a topic according to its word frequency matrix [2,30,31]. However, no study directly applies the power-law distributions of word frequencies to text typology, classification, or word distributions to text genre.

**Fig 2:**
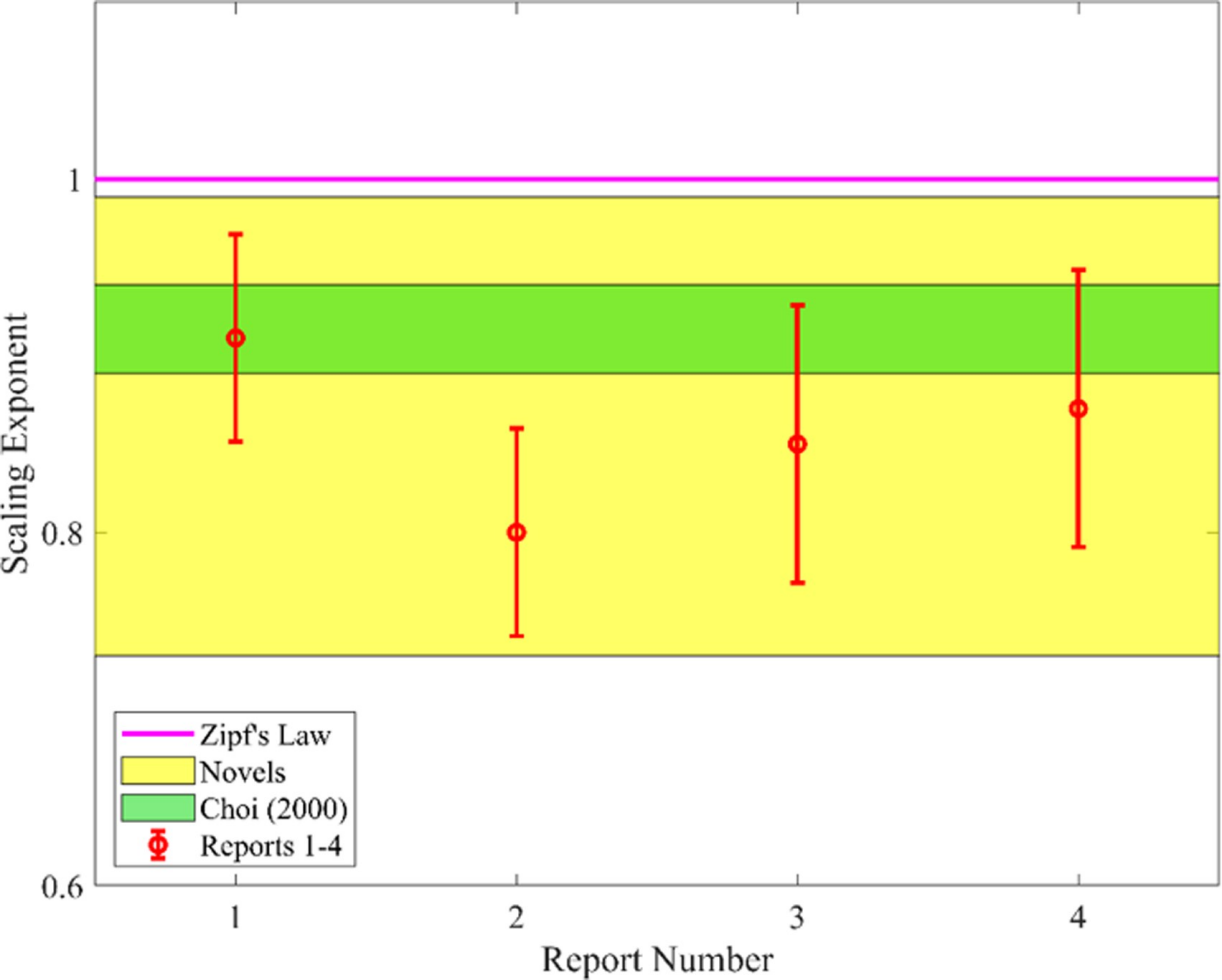
Scaling exponent *ζ* of different Korean texts. The red bars represent the scaling exponent of each event report with a 95% confidence interval. The yellow shaded area covers the 95% confidence interval of the our selected novels. The green shaded area represents the scaling exponent of Korean texts provided by Choi [22]. The pink line represents the scaling exponent of unity (*ζ* = 1), which corresponds to Zipf’s law.

We compare the scaling exponent *ζ* of different document types in Korean, as shown in Fig 2. Each 95% confidence interval of scaling exponent for event report of nuclear power plant coincides with that of three benchmark novels. Consequently, we infer that Korean documents exhibit a similar scaling exponent regardless of the document type. Therefore, the scaling behavior of word distribution alone is insufficient to identify different text genres in the Korean language. This is counter-intuitive considering the conventional knowledge that such different types of text use different vocabularies. To explain this puzzle, we introduce the interaction effect among words in a certain text as a proxy for text cohesion, functioning as an origin of this phenomenon.

### 4.3. Interaction effect

The interaction effect is defined by the rate of text cohesion and is used as a proxy for the grammatical and lexical correlations between different constituents within the text [32]. The interaction effect allows us to discern a singular piece of text against a random ensemble of unrelated sentences. Our primary measure for text cohesion is consistent with the Chomskyan position on natural language [33], utilizing adult native speakers of the according natural language as the agent of measurement. Specifically, the interaction effect is denoted as a correspondent of the discrepancy between the model-implied scaling exponent *α*_*mod*_ and its empirical counterpart *α*_*emp*_.

The controlled growth process model with interaction effects is used to characterize different genres in Korean texts, which have similar scaling exponents. Specifically, we compare the implied interaction effects of two different genres. Table 4 summarizes the estimated parameters of the controlled growth process model, including the implied interaction effect calculated from Eq (6). *α*_*mod*_, the scaling exponent obtained from the model without the interaction effect from Eq (4), exhibits a higher value than the scaling exponent directly estimated from the data, i.e., *α*_*emp*_. Therefore, we deduce the implied interaction effect *c* required to resolve this discrepancy. The interaction effects of event reports range from 0.78 to 0.90, while those of novels range from 0.28 to 0.40.

**Table 4:** Parameters of the controlled growth process model.

	*b*	*λ*	*r*	*α*_*mod*_	*α*_*emp*_	*c*
Report no. 1	0.28	0.05	0.05	2.81	1.10	0.83
Report no. 2	0.33	0.05	0.07	3.07	1.25	0.90
Report no. 3	0.33	0.06	0.07	2.71	1.18	0.84
Report no. 4	0.33	0.07	0.07	2.43	1.15	0.78
*Mu-Myeong*	0.14	0.05	0.01	1.39	1.09	0.28
*Choe-Hu-Ui-Ak-Su*	0.14	0.04	0.01	1.70	1.07	0.40
*Dong-Eop-Ja*	0.20	0.09	0.03	1.58	1.20	0.36

Note: *b* is the growth rate, *λ* is the occurrence rate, and *r* is the production rate. *α*_*mod*_ is the scaling exponent deduced from Eq (4), and *α*_*emp*_ is the scaling exponent directly estimated from the data. *c* is the model implied interaction effect.

Our results show that words from structured documents require a higher interaction effect *c* than lingual narrative literature. The interaction effect *c* is our measure for the rate of micro-level interactions between individual words within a document, i.e., “text cohesion.” This means that a technical report would exhibit higher text cohesion than unstructured text since there would be more examples of neighboring words being semantically adjacent or words from different document sections referencing each other. In contrast, a writer of the novel might utilize syntactic ambivalence as a literary device, permitting room for interpretation regarding their intended message. These intuitions coincide with the quantitive values of our metrics for interaction effects within text.

## 5. Conclusion

This study investigates the scaling exponent and text cohesion as two candidates for distinguishing Korean from other languages and distinct genres of Korean texts, respectively. Specifically, the scaling exponent is extracted by fitting word frequency data into the power-law distribution, and the controlled growth process is applied to extract the rate of text cohesion. Our results indicate that while word frequencies follow a power-law distribution, different languages show different ranges of scaling exponents according to their morphological category. Unlike English and French texts, agglutinative languages, such as Korean and Finnish, do not follow Zipf’s law. Moreover, for Korean text, the scaling exponent of experts’ reports lies within the error range of the literature, indicating that both types of documents are not significantly different in terms of scaling behavior. However, the interaction effect implied in the controlled growth process model suggests that our designated documents have a stronger text cohesion than that of novels. This supports our hypothesis that structured documents, such as expert reports, would exhibit higher text cohesion rates than general styles of texts.

Our framework can help us understand the intrinsic nature of the text, e.g., the origin of scaling behavior in the word distribution. First, a certain document has a new word whose frequency may vary within a limited range. Thus, it is more or less uniform, i.e., uniform-size production. Second, there is a tendency to reduce the frequency difference between words, which is the contribution of the interaction effect, i.e., text cohesion, to the amount of growth in word frequency. In future studies, we will apply the proposed framework to other languages and genres to establish the role of the interaction effect for different genres of texts in general.

## Supporting information

S1 Appendix Text sources. (DOCX)

S2 Appendix Parameter calibration of controlled growth process model. (DOCX)
