# Supplementary material for: Scaling behavior and text cohesion in Korean texts
Source: PLoS One. 2023 Aug 31;18(8):e0290168. doi: 10.1371/journal.pone.0290168 (PMC10470962; doi:10.1371/journal.pone.0290168)
Supplement: S1 Appendix — (DOCX) [file pone.0290168.s001.docx]

**S1 Appendix. Text sources.**

The data of event reports are provided by the Korea Institute of Nuclear Safety, and they are available to the public through <https://opis.kins.re.kr/opis?act=KROBA4100R>, which provides the Korean version of the data. Moreover, you can find the event reports written in English from <https://opis.kins.re.kr/opis?act=KEOBA4100R>, where only a few documents are available. Text files for all three novels can be freely downloaded at <https://gongu.copyright.or.kr/>, which is governed by the Korea Copyright Commission. We present the detailed source for each novel in Table A1.

**Table A1. URLs for the novels.**

| Novel name | URL |
| --- | --- |
| *Mu-Myeong* | <https://gongu.copyright.or.kr/gongu/wrt/wrt/view.do?wrtSn=13313894&menuNo=200019> |
| *Dong-Eop-Ja* | <https://gongu.copyright.or.kr/gongu/wrt/wrt/view.do?wrtSn=9000085&menuNo=200019> |
| *Choe-Hu-Ui-Ak-Su* | <https://gongu.copyright.or.kr/gongu/wrt/wrt/view.do?wrtSn=9031520&menuNo=200019> |
