# Supplementary material for: Scaling behavior and text cohesion in Korean texts
Source: PLoS One. 2023 Aug 31;18(8):e0290168. doi: 10.1371/journal.pone.0290168 (PMC10470962; doi:10.1371/journal.pone.0290168)
Supplement: S2 Appendix — (DOCX) [file pone.0290168.s002.docx]

**S2 Appendix. Parameter calibration of controlled growth process model.**

The controlled growth process model depicts the time evolution of elements forming a probability distribution. In the case of modeling the evolution of words, we considered the appearance of 100 words (previously, it was a page) as unit progression. In each unit progression, new words can emerge, and some words can reappear. Likewise, each unit progression will either create new elements or add count to existing elements. We captured the properties of this evolution process with $b$ (growth factor), $\lambda$ (occurrence rate), and $r$ (production rate). After preparing the cumulative word and its frequency list of all progressions, we estimated the parameters from the list.

First, the growth factor, $b$, is the ratio of frequency change against the previous frequency:

$$b=(x^{'}-x)/x$$

where $x$ and $x^{'}$ are the previous and updated word frequencies, respectively. Then, for each new unit progression, the growth factors for all words were calculated. Next, with all growth factors for each word throughout the document, we deduced a representative growth factor for each document. Specifically, we selected the median value of all growth factors as the representative of $b$.

Second, the occurrence rate, $\lambda$, is the rate of updates for each word frequency in our case and is defined as:

$$\lambda=l/N$$

where $l$ is the number of updated words and $N$ is the (cumulative) number of different words, which we track for each unit progression. For each new progression, some words’ frequencies can be changed while others stay the same. Therefore, when a new unit progression appears, we track whether each word frequency from the previous state has changed. Then, we calculate the occurrence rate throughout the document, and from these values, we select the median value of all occurrence rates as the representative of $\lambda$.

Finally, the production rate, $r$, is the rate of the total number of different words’ increments, which can be calibrated following Choi et al. [17]:

$$dN/dt=rN,$$

which is equivalent to the equation of $\ln N=rt+c$. Thus, we fit the value of $\ln N$ to the sequence of each unit progression ($t$) with the OLS estimator, and get its regression coefficient represents $r$ for a given document.

We should note that the number of different words does not grow exponentially for text progression. Specifically, the marginal increase in $N$ decreases as a text grows to its progression. For more practical and precise modeling of $f\left( x,t \right)$ with the nonlinear feature of $\ln N$ against $t$, we could solve the master equation numerically. However, the present study focuses on the theoretical aspect, explaining how skew distributions emerge in evolving systems by solving a master equation analytically. Accordingly, our model assumes a constant production rate for each document, and the current calibration for $r$ can represent the production rates of sample documents.
